# Systematic Review of Evening Primrose (*Oenothera biennis*) Preparations for the Facilitation of Parturition

**DOI:** 10.3390/pharmacy10060172

**Published:** 2022-12-10

**Authors:** Timothy C. Hutcherson, Nicole E. Cieri-Hutcherson, Maggie M. Lycouras, Dharmista Koehler, Madison Mortimer, Christina J. Schaefer, Olivia S. Costa, Ashley L. Bohlmann, Mudit K. Singhal

**Affiliations:** 1 Department of Pharmacy Practice, School of Pharmacy, D’Youville University, Buffalo, NY 14201, USA; 2 Department of Pharmacy Practice, School of Pharmacy and Pharmaceutical Sciences, University at Buffalo, Buffalo, NY 14214, USA; 3 Department of Pharmacy, Mount Saint Mary’s Hospital, Lewiston, NY 14092, USA; 4 The Janssen Pharmaceuticals Companies of Johnson & Johnson, Albany, NY 19044, USA; 5 Department of Pharmacy, Rochester General Hospital, Rochester, NY 14621, USA; 6 Department of Pharmaceutical, Social and Administrative Sciences, School of Pharmacy, D’Youville University, Buffalo, NY 14201, USA

**Keywords:** evening primrose, pregnancy, parturition, labor, induction

## Abstract

Background: The objective of this systematic review was to characterize the efficacy and safety of evening primrose (EP) for facilitation of parturition in peripartum persons. Methods: This search sought records related to the efficacy and safety of EP preparations to facilitate parturition. Eligibility criteria were primary literature with efficacy or safety outcomes reported; studied in peripartum persons; and available in English. Records were excluded if they were available as abstracts only. Data was synthesized by study characteristics, patient demographics, and outcomes. The RoB2 and ROBINS-I were used to assess risk of bias. Results: A total of 11 studies met inclusion criteria: seven randomized placebo-controlled trials, one randomized non placebo-controlled trial, one case study, one observational retrospective study, and one quasi-experimental cross-sectional study. Efficacy outcomes included Bishop scores and duration of labor during the different phases. Reported adverse events were generally mild and included increased blood pressure, decreased heart rate, pain, bleeding, nausea, and vomiting. Important risks of bias exist across the literature reviewed. Conclusions: The use of EP for parturition in peripartum individuals is not recommended. Further research is warranted before use during parturition or the peripartum period. Other: The authors deny conflicts of interest. The study was neither registered nor funded.

## 1. Introduction

Induction of labor is common in the United States (US), with overall frequency approaching 30% as of 2019 [1]. Per the American College of Obstetricians and Gynecologists (ACOG), induction should be considered in late-term and post-term pregnancies due to an increased risk of maternal and neonatal morbidity and mortality prior to and after birth [2]. Induction of labor is generally not recommended before 39 weeks gestation due to increased neonatal morbidity and healthcare use during the first year of life, with some exceptions. The benefits of labor induction are also weighed against the risks [3]. Potential benefits of labor induction include decreased risk of cesarean section, preeclampsia, macrosomia, and stillbirth [4]. Conversely, induction is contraindicated in patients with a history of classical or high-risk cesarean section, uterine rupture, transmural incision entering the uterine cavity, active genital herpes infection, placental previa, umbilical cord prolapse, transverse fetal lie, invasive cervical cancer, or category III fetal heart rate tracing due to associated risks [5]. Induction of labor may involve cervical ripening, also known as cervical softening, which leads to cervical effacement and dilation, ultimately allowing vaginal birth to take place.

The readiness of the cervix for labor can be assessed by the Bishop Score. A score of six or greater correlates to successful labor induction in vaginal delivery and a score of three or less correlates with a high rate of failed induction and cesarean delivery. There are numerous factors which affect the score, including: dilation, effacement, consistency, position of the cervix, and fetal station. Cervical dilation is considered most important for predicting a successful induction [6]. Other non-cervical factors that decrease the duration of induction and increase the rate of vaginal delivery include multiparity, ruptured membranes, low body mass index, tall height, low estimated fetal weight, along with the absence of preeclampsia or other comorbidities associated with placental insufficiency [7]. When the Bishop score is less than six it is recommended that a cervical ripening agent be used prior to induction of labor to better prepare the cervix for a safe birth [8].

Pharmacologic and non-pharmacologic options are available to assist in cervical ripening. An ideal ripening agent is one which is non-invasive, exerts its effects rapidly, and does not increase maternal or fetal mortality [5]. Effective methods for cervical ripening and labor induction include the use of mechanical cervical dilators, synthetic prostaglandins, and oxytocin. Natural products have also been used for parturition, including evening primrose (EP), black haw, black cohosh, blue cohosh, and red raspberry leaf. A large percentage of patients may attempt to induce labor in late pregnancy using herbal products. McFarlin and colleagues conducted a national survey of 500 members of the American College of Nurse-Midwives to characterize the use of herbal preparations for cervical ripening, induction of labor, and augmentation of labor. Herbal products for labor were used by 90 respondents and not used by 82 respondents. Of the 90 respondents that used herbal products 64% used blue cohosh, 45% used black cohosh, 63% used red raspberry leaf, 93% used castor oil, and 60% used evening primrose oil (EPO). Respondents who reported use of these products considered them natural and safe. The survey did not ascertain the participants’ rationale as to why they felt the products were safe. The respondents who reported they did not use these products stated that they were uncomfortable with the limited research available to support their use. Authors of this study concluded that there is a lack of scientific data available to support the use of these products during pregnancy. Much of their current use is based on word-of-mouth communication between providers instead of reputable scientific data [9].

The oil of EP (EPO) is extracted from the seed of the plant and contains omega-6 essential fatty acids, linolenic acid (LA) and gamma-linolenic acid (GLA). When consumed orally or administered vaginally, GLA is converted to dihomo-gamma-linolenic acid (DGLA), a precursor for prostaglandin E1 (PGE1) and thromboxane A1 [10]. It is hypothesized that EPO can increase the production of prostaglandins resulting in cervical ripening. Evidence suggests that EPO may soften the cervix, prevent post term pregnancy, and shorten the length of labor [11].

A study conducted by Degolier and colleagues examined the impact of aqueous EP extracts on mouse uterine tissue and rat cervical tissue. Aqueous EP extracts caused an increase in the force of uterine contractions in mice. Pre-contracted cervical tissue of rats was unresponsive when exposed to the EP extract. The study concluded that uterine contractions may be caused by the water-soluble components of EP and cervical ripening or relaxation may be caused by the lipid-soluble EPO [12]. According to a study done by Leaver et al., Wistar rats were given either fish oil, EPO, or a pellet breeding diet from three weeks old to at least five weeks before mating. The results showed no significant difference in parturition, birth weight, postnatal growth rate, and fetal or placental prostaglandin E2 levels between the two groups [13].

Despite the studied effects of EPO for cervical ripening, no large clinical studies clearly and definitively document the efficacy or safety of EP during parturition. EPO comes in liquid oil and capsule formulations ranging from 100 mg to 1350 mg. EPO products used in clinical studies have contained 8% to 10.5% GLA, 70% to 75% LA, 5% to 6.5% palmitic acid, 6.4% to 11.8% oleic acid, and 1.8% to 2% stearic acid. The typical oral dose is six grams daily for short-term use; however, there are no guideline-recommended topical or vaginal dosing that exist due to limited data. A study conducted by Guivernau and colleagues included healthy volunteers that received three grams of EPO as a single oral dose [14]. Plasma levels of GLA peaked in 2.7 to 4.4 h. Absorption, distribution in tissues, metabolism, and elimination has not been evaluated for vaginal or topical use of EPO. Regarding vaginal use, the optimal environment surrounding the cervical tissue, such as pH, has not been evaluated. Possible adverse reactions of oral use include flatulence, heartburn, and stomach upset. EP should be avoided in patients who have bleeding disorders, are on anticoagulants or antiplatelets, have epilepsy or seizure disorders, have schizophrenia, or in patients having surgery within two weeks. There is some evidence that EPO can decrease platelet aggregation and prolong bleeding time.

EP is a natural product which has been widely used by nurse-midwives to facilitate parturition. Despite this, there is a lack of consensus regarding evidence-based recommendations for the use of EP in this setting [15]. There have been numerous studies which sought an answer to the question of whether EP is an appropriate product to be used during parturition. The objective of this systematic review was to characterize the literature reporting on the efficacy and safety of EPO for the facilitation of parturition in peripartum persons.

## 2. Materials and Methods

### 2.1. Search Strategy

**Figure 1:**
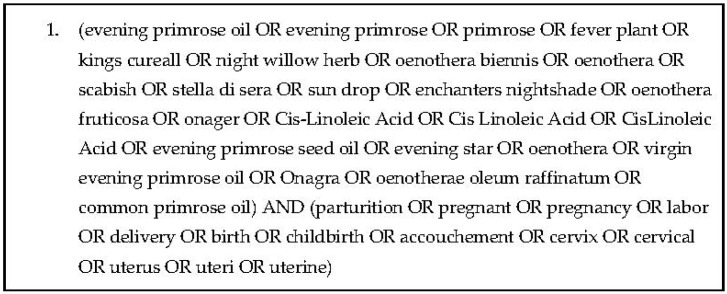
Search Strategy.

Study methods were developed according to Preferred Reporting Items for Systematic Reviews and Meta-Analyses (PRISMA) [16]. Medline, International Pharmaceutical Abstracts (IPA), Embase, Science Direct, and the Cumulative Index of Nursing and Allied Health Literature (CINAHL) were searched on 12 April 2022, seeking records regarding EP preparations and parturition. Study authors were not contacted for additional data. Medline search terms and syntax may be seen in Figure 1.

### 2.2. Study Selection

Records were independently screened by title and abstract by two reviewers; discordance was officiated by a third reviewer. Eligibility criteria included records reporting efficacy or safety outcomes for EP preparations in facilitation of parturition; studied in peripartum individuals; identified as published primary literature; and full records published in English. Abstract-only records were excluded. Citation searches of eligible records confirmed the rigor of the search strategy using the same screening and discordance officiating process previously described.

### 2.3. Data Extraction

Extraction included the characteristics and outcomes of eligible records, including: data needed to verify eligibility; unique study identifiers; data needed for risk-of-bias (RoB) assessments; methodology; study interventions, comparators, and controls; participant demographic data; and objective-specific efficacy and safety outcomes. Two reviewers independently extracted the data; discordance was officiated by a third reviewer. Outcome-compatible results were included. 

### 2.4. Data Synthesis

Data synthesis included extracted safety or efficacy outcomes from eligible studies. Data were compiled by outcome and were reported as defined in the associated study. Studies failing to report on a specific outcome were excluded from the data synthesis for that specific outcome only. Tables were created to compare study designs; patient demographics and assigned treatments; and efficacy and safety outcomes. 

### 2.5. Risk-of-Bias Assessment

RoB tools were used to evaluate eligible records based on study design; specifically, the revised Cochrane risk-of-bias tool for randomized studies (RoB2) and the Risk of Bias in Non-Randomized Studies of Interventions (ROBINS-I) [17,18]. The RoB2 tool assesses bias due to randomization, intervention deviations, missing data, outcome measures, and results selection. The ROBINS-I tool assesses bias due to confounding, intervention classification, intervention deviations, missing data, outcome measures, and reporting. RoB assessments were independently conducted by two reviewers; discordance was officiated by a third reviewer. RoB assessment was performed at the study level and then tabulated across the eligible records. 

## 3. Results

### 3.1. Search Results

**Figure 2:**
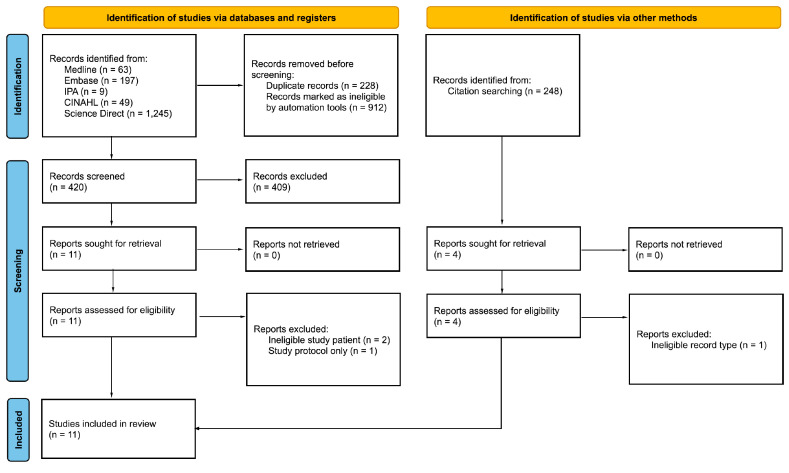
Screening and eligibility process.

The initial search of Medline, Embase, and IPA returned 259 records which were narrowed to 48 following deduplication and applying limitations of English language and excluding review articles. An additional search of the CINAHL initially returned 49 records, with 48 being deemed eligible for further review after limiting to English language. A search of ScienceDirect was conducted which returned 1245 records. Limitations of research articles, medicine and dentistry, pharmacology, toxicology and pharmaceutical science, and nursing and health professions were applied, leaving 336 records eligible for further review. After initial screening 15 potential records remained, 11 of which met eligibility criteria (Figure 2) and were included in this review. Evaluated studies included seven randomized placebo-controlled trials, one randomized non placebo-controlled trial, one case study, one observational retrospective study, and one quasi-experimental cross-sectional study [19,20,21,22,23,24,25,26,27,28,29]. 

Resultant records included seven randomized placebo-controlled trials, one randomized non placebo-controlled trial, one case study, one observational retrospective study, and one quasi-experimental cross-sectional study that assessed the effect of EPO alone or in combination with other supplements or medications in peripartum persons during parturition [19,20,21,22,23,24,25,26,27,28,29]. The type of intervention and control (if used), study design, population, study location, and duration are reported in Table 1.

Dove and Johnson (1999) conducted a single-center, quasi-experimental, superiority study designed to investigate the effects of EP on the length of pregnancy and pregnancy outcomes in low-risk nulliparous peripartum persons [19]. The study took place from January 1991 to September 1998. Included were individuals who had accurate gestational dating, cephalic presentation, low-risk status, and delivered between 38 and 42 weeks gestation. There were no exclusion criteria listed due to the retrospective nature of this study. The primary outcome was mean time in labor, with results reported in Table 2. Safety outcomes were not reported in this study. Authors concluded that EPO does not shorten the length of labor or gestation. 

The case report authored by Wedig et al., (2008) described a healthy, 31-year-old who was given oxytocin and gave birth to a female who was born at 38 weeks and four days gestation weighing 2885 g [20]. This case report provides relevant safety data, reported in Table 3. The infant was born after 16 h of labor and had Apgar scores of nine. Diffuse ecchymoses and petechiae appeared on the neonate’s trunk, extremities, and face at 17 h of age. A physical examination was unremarkable except for mild jaundice. She was transferred to the neonatal intensive care unit (NICU) for observation, where platelet count, head ultrasound, and urinalysis were normal. In the NICU, no new ecchymoses were seen and she was discharged at five days of age. The authors concluded that EPO has not been proven safe and effective and should be further studied.

Zahran et al., (2009) conducted a double-blind, superiority randomized controlled trial (RCT) which sought to determine whether outpatient administration of EPO was a safe and effective method of labor induction in women with postdate pregnancy [21]. The study took place at the Women’s Health Centre of Assiut University from January 2008 to July 2009. Patients were included if they had a gestational age of at least 40 weeks, without an urgent indication for labor induction, a singleton living fetus with average amniotic fluid and a non-reactive stress test with Bishop score eight or less. Excluded were those with medical or obstetric indications for termination, fetuses larger than 4000 g, presence of fetal anomalies, intrauterine growth retardation, previous uterine scarring, premature membrane rupture, cephalopelvic disproportion, and presence of any other contraindications to vaginal delivery. The primary outcome was frequency of successful induction, with a secondary outcome measure being a change in Bishop score from admission to labor. Results of the secondary outcome are reported in Table 2. Regarding the primary outcome, frequency of successful induction defined as birth within 14 days of drug administration, the mean number of days from drug use to birth was 7.4 ± 1.2 and 8.4 ± 1.4 for the EP and placebo groups, respectively. Safety data is reported in Table 3. Authors of the study concluded that EPO is well tolerated in this patient population with negligible complications, but that it was not beneficial in shortening gestation or improving the Bishop score. 

Jahdi et al., (2016) conducted a triple-blind, superiority RCT to determine the effectiveness of oral EPO on the ripening and softening of the cervix in nulliparous peripartum persons [22]. The study was conducted from August to December 2015. Included were healthy, low-risk peripartum persons from Iran with a gestational age of between 40 weeks to 40 weeks and six days, a Bishop score less than 4, a healthy membrane, no drug use, and those who avoided intercourse and did not use enemas, laxatives, or herbals during the study. Exclusion criteria were not listed. The primary outcome was the Bishop score, with results reported in Table 2. Safety data was not reported in this study. Authors concluded that taking one EPO capsule every twelve hours did not have a statistically significant difference on the Bishop score.

Diansuy N et al., (2017) conducted a cross-sectional quasi-experimental study to determine the efficacy of EPO capsules for cervical ripening amid induction of labor [23]. The study was conducted from May to July of 2016. Inclusion patients were 18 years or older with accurate gestational age, singleton term pregnancy, cephalic presentation, Bishop score of four or less, intact amniotic membranes, biophysical profile of 100%, and those with stable maternal conditions. Excluded were those with contraindications to vaginal delivery due to placenta previa, previous uterine scarring, or an estimated fetal weight greater than or equal to 4000 g. The primary outcome was the Bishop score, with results reported in Table 2. Safety data was not reported on in this study. Authors concluded that using vaginal EPO in singleton pregnant peripartum persons was promising for cervical ripening, but additional research was warranted.

Kalati et al., (2018) conducted a triple-blind, single center, superiority RCT to evaluate the effectiveness of EPO on the duration of pregnancy and labor [24]. The study was conducted from March 2014 to August 2015. Inclusion criteria were nulliparous Iranian individuals at least 40 weeks gestation with a low-risk singleton pregnancy, cephalic presentation, Bishop score less than 4, BMI between 19–25 kg/m^2^ and an estimated fetal weight of 2500 to 4000 g. Excluded were those with high risk pregnancies, complications during pregnancy; presence of serious maternal systemic disorders; history of drug use; vaginal bleeding; fetal distress; ruptured membranes; use of other methods for cervical ripening; or development of adverse effects from the study drug. The primary outcome was the Bishop score, with results reported in Table 2. Safety data is reported in Table 3. Authors concluded that there is insufficient evidence to support the effectiveness of EPO for cervical ripening.

Najafi et al., (2019) conducted a double-blind, single center, superiority RCT designed to determine the impact of vaginal EP capsules on the Bishop score in nulliparous peripartum persons at term [25]. The study was conducted from November 2017 to May 2018. Patients were included if they were between 18–35 years of age and had a vaginal delivery, at least 38 weeks gestation, nulliparous with an intact amniotic sac, and a singleton fetus with a live and healthy embryo. Excluded were those not using a vaginal EP capsule for two consecutive times, allergic to the capsule, with an indication for urgent medical intervention for maternal or fetal needs, use of enemas, laxatives, or herbal capsules, or patients engaging in intercourse to facilitate delivery. The primary outcome was the change in Bishop score during the stages of labor, with results reported in Table 2. Safety outcomes are reported in Table 3. Authors concluded that use of vaginal EP capsules may be an effective, safe, and affordable way to facilitate cervical ripening in nulliparous peripartum persons at full term. 

Hashemnejad et al., (2019) conducted a triple-blind, single-center, superiority RCT to investigate the effectiveness of vaginal administration of EP in inducing delivery [26]. The study was conducted during 2019. Inclusion criteria were peripartum persons at 37 weeks gestation in patients who had no previous hospitalizations prior to giving birth, Bishop score less than four, without an indication for emergency delivery. Excluded were who declined to participate in the study for any reason. The primary outcome measures were delivery pain commencement, labor duration, and delivery time. Mean interval between administration and onset of pain were 19.06 and 13.4 h for the EP and control groups, respectively. The mean interval between administration and delivery were 73.83 and 60.68 h, respectively. Safety data was not mentioned in the study. Authors concluded that vaginal administration of EP capsules was not effective in cervix preparation. Authors noted that further research is needed to adequately determine the effects of EPO. 

Bahmani et al., (2019) conducted a single-blind, single center, superiority RCT to compare the effects of vaginal EPO capsules and vaginal misoprostol on cervical ripening in nulliparous peripartum persons, during post-term pregnancy [27]. This study was conducted in 2018. Inclusion were healthy, willing, and nulliparous individuals with a single pregnancy, 40 weeks and six days gestational age, with no contraindications to EPO or misoprostol, no structural cervical abnormalities, Bishop score of four or less, and presence of a live fetus. Excluded from the study were those who used enemas, laxatives, or herbal medicines prior to the study, those unwilling to cooperate at any point, need for cesarean section, or the development of adverse effects from the study drug. The primary outcome measure was the Bishop score, with the results reported in Table 2. Safety data was not mentioned in the study. Authors concluded that use of vaginal EPO with misoprostol was more effective in reducing the Bishop score and improving cervical ripening in this population, compared to the use of misoprostol alone. 

Mirzadeh et al., (2020) conducted a single-center, single-blind, superiority RCT comparing the effects of vaginal misoprostol tablets with vaginal EP capsules in cervical ripening in nulliparous peripartum persons [28]. The study was conducted from April to November 2018. Peripartum individuals aged 18–30 years old with a low-risk first pregnancy and a gestational age of 40 weeks to 40 weeks and six days, live cephalic fetus, Bishop score less than four, intact amniotic sac, and estimated fetal weight of 2500 to 4000 g. Excluded from the study were those with a vaginal exam 24 h prior to the study, used other herbal medication, or engagement in sexual intercourse during the study. The primary outcomes were mean Bishop score at admission and comparison of the Bishop score components at admission, with results reported in Table 2. Cervical dilatation and consistency at time of admission were significantly improved in the EP group (*p* < 0.001). Safety data is reported in Table 3. Authors of this study concluded that vaginal EP capsules could be effective regarding cervical ripening and dilatation. 

Azad et al. (2022), performed a single-center, double-blind, superiority RCT to assess the effects of EPO on ripening of the cervix in post-term pregnant women [29]. The study occurred between November 2018 to December 2019. Included in the study were peripartum individuals aged 18–35 years during their first pregnancy with a live singleton baby; gestational age of 41 weeks or greater; cephalic presentation with intact membranes; and a Bishop score of four or less. All cases of major fetal anomaly, fetal growth restriction, fetal distress, uterine anomaly, abnormal vaginal bleeding, or history of uterine surgery were excluded from the study. The primary outcome was the Bishop score, with results reported in Table 2. Safety data is reported in Table 3. Authors of this study concluded that vaginally administering a single 1000 mg dose of EPO at 41 weeks gestation significantly improved the Bishop score, as well as reduced post-term parturition time. Authors noted that further studies would help optimize EPO dosing for cervical ripening.

### 3.2. Risk-of-Bias Results

The following limitations were identified across the eligible records: non-randomized studies (n = 3) and insufficient randomization (n = 3); concurrent drug use not discussed (n = 1); identifying onset of labor not discussed (n = 2); length of study (n = 1); insufficient blinding (n = 3); small sample size, power not met, and no statistically significant results for primary outcome measures (n = 2); incorrect administration of medications (n = 1). Risk-of-bias assessments were conducted for each of the eleven records (see Table 4). The studies conducted by Zahran et al. (2009), Kalati et al. (2018), Najafi et al. (2019), Jahdi et al. (2016), Hashemnejad et al. (2019), and Azad et al. (2022) had low risk-of-bias overall in all domains [21,22,24,25,26,29]. Risk-of-bias was identified in both the Mirzadeh et al. (2020) and Bahmani et al. (2018) studies, as investigators were unblinded [27,28]. Diansuy et al. (2017) had a critical risk-of-bias identified due to the presence of a confounding variable and the selection of participants was favored having only 13 participants and no control group [23]. Dove et al. (1999) had a critical risk-of-bias due to its retrospective study design which allowed them to select participants after the end of treatment [19]. Wedig et al. (2008) had low risk-of-bias in all domains and for the study overall [20]. 

## 4. Discussion

This study was conducted to characterize the available literature concerning the safety and efficacy of EP products during parturition. Many of the studies used the Bishop score and duration of time per stage of labor to measure effectiveness [19]. Other outcomes that were not universal among the studies included: the type of delivery, Apgar scores, and use of oxytocin for delivery [19,21,24]. There was a wide range of variability regarding the efficacy results. The outcome measures reported were valid and comprehensive among the studies; however, the Bishop score is the gold standard assessment tool that predicts the readiness of the cervix for successful induction of labor [6].

There were a variety of safety outcomes reported by the included studies; most were considered mild, tolerable, and did not lead to discontinuation of the study drug or major changes to course of care. Long-term safety outcomes were not assessed in any of the studies. Three studies reported that no side effects occurred in participants [19,23,24]. In the case study, the newborn had diffuse ecchymoses and petechiae on the trunk, extremities, and face after vaginal and oral administration of EP [20]. Three studies did not report on safety outcomes [19,22,25]. Since contraindications for use of EP include individuals with bleeding disorders or concomitant administration of anticoagulant or antiplatelet medications, the side effects reported in the case report may be particularly important to note [14,20]. EPO has also been shown to inhibit platelet aggregation due to the presence of dihydrolipoic acid which may explain an increased risk of bleeding [20]. Due to these safety considerations, EPO cannot be recommended as benign or safe, which is a common misconception among laypersons regarding the use of herbal products.

Dose and route of administration varied among the studies. Typical dosages ranged from 500 mg by mouth or vaginally once to three times a day, 1000 mg by mouth or intravaginally once or twice daily, or two 1000 mg capsules intravaginally every 12 h. Appropriate and complete administration was observed in most studies except for Jahdi et al. and Kalati et al. [24,26]. EPO products used in clinical studies have contained 8% to 10.5% GLA, 70% to 75% LA, 5% to 6.5% palmitic acid, 6.4% to 11.8% oleic acid, and 1.8% to 2% stearic acid [14]. EPO is available in both capsule and liquid formulations. The commercial preparation of the specific capsule product Efamol EPO contains 720 mg of LA, 80 mg of GLA, and trace amounts of oleic, stearic, and palmitic acid [10,15]. The average cost for a single 500 mg EP capsule is $0.07, red raspberry leaf capsules cost $0.50 per capsule and misoprostol costs between $0.82 to $1.43 per tablet [30]. Further research is needed to provide supporting evidence for use and ideal dose and route of administration for EPO. No information exists for absorption, distribution, metabolism, or elimination of EPO when administered intravaginally [31]. In a study of healthy volunteers, a three-gram dose of EPO administered orally produced peak plasma levels of GLA between 2.7 to 4.4 h on average. 

Additionally, the route of administration should be considered prior to making therapeutic recommendations. Inserting EPO vaginally may be uncomfortable for the patient, and it may lead to oily discharge. There is insufficient information available regarding the clinical presentation or treatment of EP overdose. Many of the reviewed studies administered EPO, vaginally or orally, for a duration between 1–3 weeks at variable doses. Participants did not experience any significant side effects related to EPO. EPO results in high levels of GLA in breast milk, which is a normal component found in breast milk regardless of EPO use [32].

A universal feature of the reviewed studies was their small sample size. Despite this, four studies were adequately powered for their outcomes [21,24,25,26]. Two studies were unable to meet power and did not have a statistically significant outcome [22,28]. The study locations varied, which could have negatively impacted the results due to the high level of variability within each patient population. Different countries vary in their practice of medicine and standards of care. On average, the duration of the studies lasted seven days, but it is unknown if one week is sufficient to prove efficacy or safety of EPO for the facilitation of parturition. Overall, all studies reviewed, apart from Diansuy et al., Dove et al., and Wedig et al., were appropriately randomized to produce similar comparator groups [19,20,23]. When a control group was used, the products were similar in shape and size to the study drug, and the dosing frequency and duration of treatment were the same. Raspberry leaf capsules were used in Wedig et al., but no other studies discussed additional herbal product usage [20].

An important inclusion criterion in all reviewed studies was that pregnant individuals were at least 37 weeks gestation with a viable fetus. This was important because induction is not recommended prior to 37 weeks, or in the case of a non-viable fetus [19,20,21,22,23,24,25,26,27,28,29]. Seven studies required a Bishop score of four or less; [22,23,24,26,27,28,29] three studies did not consider the Bishop score as an inclusion criteria [19,20,25]. Seven studies required participants to be at a low risk for pregnancy or otherwise healthy; [19,20] six studies required pregnant participants to be nulliparous as a factor for inclusion [22,24,25,27,28,29]. Due to a lack of standardization among the inclusion criteria, baseline characteristics are highly variable across the studies, introducing bias.

A common exclusion criterion among the studies was pregnant individuals who anticipated or underwent urgent medical interventions during the study. Certain comorbidities, such as preeclampsia and gestational diabetes, may increase maternal morbidity and cause complications during childbirth. The use of other methods for cervical ripening, such as other herbal medicines or sexual intercourse, was an exclusion criterion in four of the studies [24,25,27,28]. This was a valuable exclusion criterion as it allowed study authors to assess the effects of EP without confounding variables. This may be helpful for future studies to bolster the evidence for or against EPO monotherapy. 

A variety of conclusions were made by the study authors regarding efficacy and safety of EPO for cervical ripening. Most authors suggested further research was needed due to the lack of supporting the effectiveness of EPO in facilitating parturition [19,20,21,22,24,26]. Three of these six studies explicitly stated that EPO was not effective in improving Bishop score or preparation of the cervix for induction of labor [21,22,26]. Diansuy et al. showed promise in using EPO for cervical ripening, however further research was required to determine its effectiveness [23]. Mirzadeh et al. and Najafi et al. concluded that vaginal EPO could be effective for cervical ripening [25,28]. The data in Bahmani et al. supported the conclusion that vaginal EPO used with misoprostol was more effective on Bishop score and cervical ripening vs. monotherapy with misoprostol [27]. Azad et al. concluded that vaginal EPO improved Bishop scores and decreased parturition time for individuals with post-date pregnancies [29]. Despite the limited data supporting its efficacy and safety, EPO continues to be utilized for cervical ripening [25]. 

In the US, herbal products are labeled as dietary supplements and are not approved by the US Food and Drug Administration (FDA). There are many factors that contribute to the quality of herbal medicines; however, their complexity poses challenges in determining purity, efficacy, and safety of the product [9]. Quality issues of herbal products can be associated with the quality of the raw materials and finished products. Contaminants like metals, pesticides, microbes, and other foreign matter may cause serious harm. Adding improper or inferior ingredients like metals or sands would likely affect purity, efficacy, and safety. Ingredients that are additive or non-uniform introduce impurities, and they can potentially alter the therapeutic effect of the final product [33]. This is particularly concerning as it related to the use of herbal products during pregnancy.

EPO is absent from all relevant guidelines, such as the ACOG, the Society of Maternal-Fetal Medicine, and the American Society for Reproductive Medicine [2,34,35]. The overall efficacy of EPO needs to be further evaluated due to the conflicting results that were determined in studies that have been previously conducted and included in this analysis. Additionally, a more thorough review of safety outcomes in intravaginally administered EPO is warranted. Limited data exists regarding long-term maternal and neonatal safety outcomes, maternal recovery time, ease of delivery, and EPO levels present in breast milk. It is important to consider the impact to the fetus or infant as a result of administration of medications and natural products. A risk versus benefit discussion will be essential between the provider and the patient regarding the use of EPO for this indication.

A limitation of this systematic review is a lack of consistent data regarding the use of EPO for the facilitation of parturition. While compiling the relevant information, it was discovered that there was much heterogeneity in the safety and efficacy data, making it difficult to draw any strong conclusions. Additionally, the search strategy was designed to be comprehensive, but several indexing or tagging errors in the literature which may have led to the inappropriate exclusion of relevant records. Finally, every piece of existing literature was unable to be assessed as there were some written in other languages and not published or adapted into English. Therefore, it is possible that there is a gap in the data presented which will need to be reexamined in the future if those pieces of data become available to us. 

## 5. Conclusions

The objective of this systematic review was to characterize the literature on the efficacy and safety of EPO and related preparations for the facilitation of parturition in peripartum persons. Based on the limited data available, EPO cannot be recommended for parturition in peripartum individuals based on the principle of “do no harm”. There is a need for further research to evaluate safety and efficacy of EPO during pregnancy due to its continued frequent use. Alternatively, education of providers about the questionable efficacy and the safety concerns of EPO could help to limit its use.

## Figures and Tables

**Table 1:** Included studies with select design characteristics.

Authors (Year)	Design (Partici-Pants)	Population, Study Location	Intervention	Control	Duration
Dove et al. (1999) [19]	Retrospective quasi-experimental study (n = 108)	Peripartum persons, United States	EP 500 mg capsule by mouth 3 times daily for 1 week at 37 weeks gestation, then 500 mg once daily until delivery	No EP	37 weeks gestation until delivery
Wedig et al. (2008) [20]	Case study (n = 1)	31 year old Female	EP 500 mg 13 capsules total vaginally and orally	N/A	N/A
Zahran et al. (2009) [21]	RCT (n = 240)	Peripartum persons, Egypt	EP 1000 mcg capsules by mouth every 12 h for up to 10 days	Placebo	10 days
Jahdi et al. (2016) [22]	RCT (n = 80)	Peripartum persons, Iran	EP 1000 mg capsule by mouth every 12 h for 7 days	Placebo	7 days
Diansuy et al. (2017) [23]	Quasi-experimental study (n = 13)	Peripartum persons, Philippines	EP 1000 mg 2 capsules vaginally once	None	Day of labor
Bahmani et al. (2018) [27]	RCT (n = 130)	Peripartum persons, Iran	EP 500 mg capsule vaginally and misoprostol 25 mcg SL if ineffective after 6 h 2 additional doses of each could be given	Misoprostol 25 mcg SL/Placebo	7 days
Kalati et al. (2018) [24]	RCT (n = 80)	Peripartum persons, Iran	EP 1000 mg capsule by mouth twice daily for 7 days	Placebo	7 days
Hashemnajad et al. (2019) [26]	RCT (n = 162)	Peripartum persons, Iran	EP 1000 mg 2 capsules vaginally once	Placebo	Day of labor
Najafi et al. (2019) [25]	RCT (n = 86)	Peripartum persons, Iran	EP 1000 mg capsule vaginally once daily from 38 weeks gestation until delivery	Placebo	38 weeks gestation until delivery
Mirzadeh et al. (2020) [28]	RCT (n = 100)	Peripartum persons, Iran	EP 1000 mg capsule vaginally once daily for 7 days	Misoprostol 25 mcg vaginally	7 days
Azad et al. (2022) [29]	RCT (n = 175)	Peripartum persons, Iran	EP 1000 mg (two 500 mg capsules) inserted intravaginally 6 h before labor induction with oxytocin	Placebo	Day of labor

EP: evening primrose; Mcg: micrograms; Mg: milligrams; N/A: not applicable; RCT: randomized controlled trial.

**Table 2:** Select efficacy results.

Authors (Year)	Mean Bishop Score after Intervention	Mean Time in 1st Stage of Labor (Min)	Mean Time in 2nd Stage of Labor (Min)	Mean Time in 3rd Stage of Labor (Min)	Mean Total Time in Labor (Hours)
Dove et al. (1999) [19]	NR	NR	NR	NR	EP: 15.66 ± 10.27 Placebo: 12.67 ± 6.15 (*p* = 0.002)
Zahran et al. (2009) [21]	EP (induced labor onset): 6.8 ± 1.4 Placebo (induced labor onset): 6.6 ± 1.3 *p* > 0.05 EP (spontaneous labor onset): 8.4 ± 2.2 Placebo (spontaneous labor onset): 7.4 ± 2.0 *p* > 0.05	NR	NR	NR	EP (induced labor): 14.2 ± 3.9 Placebo (induced labor): 17.8 ± 3.2 *p* > 0.05 EP (spontaneous labor): 8.2 ± 2.9 Placebo (spontaneous labor): 10.0 ± 3.5 *p* < 0.05
Jahdi et al. (2016) [22]	EP: 3.60 ± 1.75 Placebo: 4.35 ± 2.34 (*p* = 0.431)	NR	NR	NR	NR
Diansuy et al. (2017) [23]	Improvement in Bishop score (n = 11) Score of 4 or higher (n = 4)	NR	NR	NR	NR
Bahmani et al. (2018) [27]	EP and misoprostol: 5.08 ± 1.62 Placebo and misoprostol: 3.08 ± 1.72 (*p* < 0.05)	NR	NR	NR	NR
Kalati et al. (2018) [24]	EP: 3.60 ± 1.75 Placebo: 4.35 ± 2.34 (*p* = 0.110)	EP: 524.48 ± 240.21 Placebo: 530.62 ± 223.37 (*p* = 0.906)	EP: 45.75 ± 31.71 Placebo: 57.37 ± 33.12 (*p* = 0.113)	EP: 8.12 ± 5.27 Placebo: 7.50 ± 3.39 (*p* = 0.530)	EP: 9.63 ± 4.62 Placebo: 9.92 ± 4.33
Najafi et al. (2019) [25]	EP: 5.93 ± 2.42 Placebo: 2.81 ± 2.02 (*p* = 0.001)	EP: 283.55 ± 297.41 Placebo: 525.95 ± 306.95 (*p* = 0.006)	EP: 249.55 ± 131.27 Placebo: 226.52 ± 132.53 (*p* = 0.52)	EP: 54.7 ± 36.11 Placebo: 64.75 ± 43.63 (*p* = 0.36)	EP: 9.79 ± 7.75 Placebo: 13.6 ± 8.05
Mirzadeh et al. (2020) [28]	EP: 5.38 ± 0.93 Misoprostol: 5.19 ± 1.114 (*p* = 0.272)	NR	NR	NR	NR
Azad et al. (2022) [29]	EP: 6.96 ± 0.18 Placebo: 3.67 ± 0.25	EP: 220.2 ± 64.8 Placebo: 205.2 ± 69.6 (*p* = 0.244)	EP: 438.6 ± 127.8 Placebo: 588.6 ± 64.8 (*p* = 0.031)	EP: 68.31 ± 12.13 Placebo: 70.31 ± 11.03 (*p* = 0.531)	EP: 726.6 ± 67.8 Placebo: 864 ± 48 (*p* = 0.036)

EP: evening primrose; Min: minutes; NR: not reported; Wedig et al. and Hashemnajad et al. did not report data for outcomes listed in this table [20,26].

**Table 3:** Safety results listed by author (year).

Authors (Year)	Safety Results
Wedig et al. (2008) [20]	Newborn had diffuse ecchymoses and petechiae on trunk, extremities, and face
Zahran et al. (2009) [21]	Diarrhea (EP: n = 5; placebo: n = 5; *p* = NS) Nausea/vomiting (EP: n = 15; placebo: n = 12; *p* = NS) Meconium aspiration (EP: n = 3; placebo: n = 4; *p* = NS) Apgar score < 7 at 1 min (EP: n = 16; placebo: n = 14; *p* = NS) Apgar score < 7 at 5 min (EP: n = 5; placebo: n = 4; *p* = NS) NICU admissions (EP: n = 4; placebo: n = 2; *p* = NS)
Jahdi et al. (2016) [22]	Increased blood pressure (placebo: n = 1) Decreased heart rate and bleeding (placebo: n = 3)
Kalati et al. (2018) [24]	No adverse events were observed or reported in either group
Hashemnejad et al. (2019) [26]	No adverse events were observed or reported in either group
Najafi et al. (2019) [25]	Abnormal hemorrhage during first 2 h after delivery EP: n = 3 (7%) Placebo: n = 4 (9.5%) (*p* = 0.66)
Mirzadeh et al. (2020) [28]	Pain EP: n = 18 (40%) misoprostol: n = 41 (74.5%) Nausea and vomiting EP: n = 12 (26.7%) misoprostol: n = 36 (65.5%) (*p* < 0.001) Bleeding at admission EP: n = 5 (11.1%) misoprostol: n = 1 (1.8%) (*p* < 0.001)
Azad et al. (2022) [29]	No adverse events were observed or reported in either group

EP: evening primrose; NICU: neonatal intensive care unit; NS: not significant; Dove et al., Diansuy et al., and Bahmani et al., did not report data for safety outcomes [19,23,27]; Authors of Zahran et al. reported their results as either “SS” or “NS” for statistically significant and not significant, respectfully [21].

**Table 4:** Risk-of-bias results.

**Randomized Controlled Trials**								
**Authors** **(Year)**	**Overall RoB**	**Randomization**	**Outcome Deviations**	**Missing Data**	**Outcome Measures**	**Results Selection**	-	-
Zahran et al. (2009) [21]	⬤	⬤	⬤	⬤	⬤	⬤	-	-
Jahdi et al. (2016) [22]	⬤	⬤	⬤	⬤	⬤	⬤	-	-
Kalati et al. (2016) [24]	⬤	⬤	⬤	⬤	⬤	⬤	-	-
Bahmani et al. (2018) [27]	⬤	⬤	⬤	⬤	⬤	⬤	-	-
Hashemnajad et al. (2019) [26]	⬤	⬤	⬤	⬤	⬤	⬤	-	-
Najafi et al. (2019) [25]	⬤	⬤	⬤	⬤	⬤	⬤	-	-
Mirzadeh et al. (2020) [28]	⬤	⬤	⬤	⬤	⬤	⬤	-	-
Azad et al. (2022) [29]	⬤	⬤	⬤	⬤	⬤	⬤	-	-
**Non-Randomized Studies**								
**Authors** **(Year)**	**Overall RoB**	**Participant Selection**	**Intervention Deviations**	**Missing Data**	**Outcome Measures**	**Results Selection**	**Intervention Classification**	**Confounding**
Dove et al. (1999) [19]	⬤	⬤	⬤	⬤	⬤	⬤	⬤	⬤
Diansuy et al. (2017) [23]	⬤	⬤ Favors intervention	⬤	⬤	⬤	⬤	⬤	⬤
**Case Reports**								
**Authors** **(Year)**	**Overall** **RoB**	**Demographics**	**History**	**Condition**	**Outcome Measures**	**Intervention**	**Results**	**Adverse Events**
Wedig et al. (2008) [20]	⬤	⬤	⬤	⬤	⬤	⬤	⬤	⬤

Red dots incidicate of high risk of bias; yellow dots indicate unknown or intermediae risk of bias; green dots indicate low risk of bias.

## Data Availability

Not applicable.
